# Examining relationships between age at diagnosis and health-related quality of life outcomes in prostate cancer survivors

**DOI:** 10.1186/s12889-018-5976-6

**Published:** 2018-08-23

**Authors:** Christine J. Kurian, Amy E. Leader, Melissa S. Y. Thong, Scott W. Keith, Charnita M. Zeigler-Johnson

**Affiliations:** 1 Sidney Kimmel Medical College, Thomas Jefferson University, Philadelphia, PA USA; 2 Division of Population Science, Department of Medical Oncology, Sidney Kimmel Medical College, Thomas Jefferson University, Philadelphia, PA USA; 3 Department of Medical Psychology, Amsterdam University Medical Centers, location AMC, Amsterdam, The Netherlands; 4 Division of Biostatistics, Department of Pharmacology and Experimental Therapeutics, Sidney Kimmel Medical College, Thomas Jefferson University, Philadelphia, PA USA

**Keywords:** Prostate cancer, Health-related quality of life, HRQL, Age at diagnosis

## Abstract

**Background:**

Patient reports of health related quality of life can provide important information about the long-term impact of prostate cancer. Because patient symptoms and function can differ by age of the survivor, the aim of our study was to examine patient-reported quality of life and prostate symptoms by age at diagnosis among a registry of Dutch prostate cancer survivors.

**Methods:**

A population of 617 individuals from the Patient Reported Outcomes Following Initial Treatment and Long-Term Evaluation of Survivorship (PROFILES) database was surveyed using the European Organization for Research and Treatment of Cancer Quality of Life Questionnaire (EORTC-QLQ-C30) and prostate symptom (EORTC QLQ-PR25) scales. Age at diagnosis was the main independent variable, with three age categories: 60 years and younger, 61–70 years, and 71 years and older. Dependent variables were the EORTC-QLQ-C30 and EORTC QLQ-PR25 scales, divided into positive and negative outcomes. Positive measures of health-related quality of life included global health, physical functioning, role functioning, emotional functioning, cognitive functioning, and social functioning. Negative outcomes included fatigue, nausea, pain, dyspnea, insomnia, appetite, constipation, and diarrhea. We also assessed sexual activity, and urinary, bowel and hormonal symptoms. Descriptive analyses included frequencies with chi-square tests and medians with Kruskal-Wallis tests. Multivariable adjusted analyses were conducted by median regression modeling.

**Results:**

Among the numerous scales showing some unadjusted association with age group, only two scales demonstrated significant differences between prostate cancer patients age 71+ compared to the youngest group (age < 61) after multivariable adjustment. On average, the oldest patients experienced an 8.3-point lower median physical functioning score (β = − 8.3; 95% CI = − 13.9, − 2.8; *p* = 0.003) and a 16.7-point lower median sexual activity score (β = − 16.7; 95% CI = − 24.7, − 8.6; *p* < 0.001) while controlling for BMI, marital status, time since diagnosis, comorbidities (heart condition), Gleason score, and treatment (prostatectomy).

**Conclusions:**

Results suggest that patient age at diagnosis should be considered among factors that contribute to health-related quality of life outcomes for prostate cancer survivors. *Implications for Cancer Survivors*: A possible reevaluation of screening recommendations may be appropriate to acknowledge age as a factor contributing to health-related quality of life outcomes for prostate cancer survivors.

## Background

Prostate cancer is the second most common cancer worldwide in men, with approximately 70% of new cases diagnosed in 2012 found in developed countries [1]. The majority of these men, however, do not die from prostate cancer. As the number of prostate cancer survivors increases, ensuring health-related quality of life (HRQL) becomes increasingly important for better overall health outcomes.

Quality of life measures encompass the physical, emotional, and social domains [2]. HRQL in prostate cancer patients has been examined in varying capacities using diverse means of quantifying data. A cross-sectional study using the Cancer Rehabilitation Evaluation System (CARES) found that quality of life declined with increased time for prostate cancer survivors [3]. However, another study found that functional status scores did not decline within one year after diagnosis for prostate cancer [4]. Diefenbach et al. found that higher levels of functional quality of life lead to lower levels of distress for both those patients older and younger than 68 [5].

Although some studies [2] did not observe that age at diagnosis predicted quality of life, other studies suggest that age at diagnosis can have significant, varying effects on subsequent HRQL [6–8]. Whether an earlier diagnosis leads to an increased propensity for further positive lifestyle changes, or instead leads individuals to worse health outcomes, however, is a topic of debate. Lintz and colleagues found that patients diagnosed at a younger age reported increasing fatigue [6]. Pinkawa et al., evaluated quality of life measures in German prostate cancer patients before treatment [7]. This study found that increased patient age affected HRQL negatively for prostate cancer patients that were about to undergo treatment, particularly in the spheres of increasing urinary incontinence, increasing urinary bother (including irritation and obstruction), and decreasing sexual function with increasing age. Kikkawa and colleagues evaluated quality of life in Japanese prostate cancer patients specifically treated by high-dose rate brachytherapy combined with external beam radiotherapy 24 months after treatment [8]. Their study utilized the Medical Outcome Study 8-items Short Form Health Survey and the EPIC questionnaire and found that older men did not differ significantly from younger men in outcome scores from both questionnaires.This wide range of findings demonstrates the variability in relating age with quality of life in cancer patients and was thus an impetus for the study at hand. Those diagnosed at a younger age are often at a different health trajectory compared to those diagnosed in the later years of life. When cancer is diagnosed at a younger age, individuals often need to completely shift their health behaviors, specifically by adopting more prudent lifestyle habits and focusing on necessary outside resources [9]. Whether this shift reflects positively upon overall HRQL has yet to be thoroughly examined. Patient reports of HRQL can provide important information about the long-term impact of prostate cancer. Because patient symptoms and function can differ by age of the survivor, the aim of our study was to examine HRQL and prostate symptoms by age at diagnosis among a registry of Dutch prostate cancer survivors.

## Methods

### Setting and participants

**Fig. 1:**
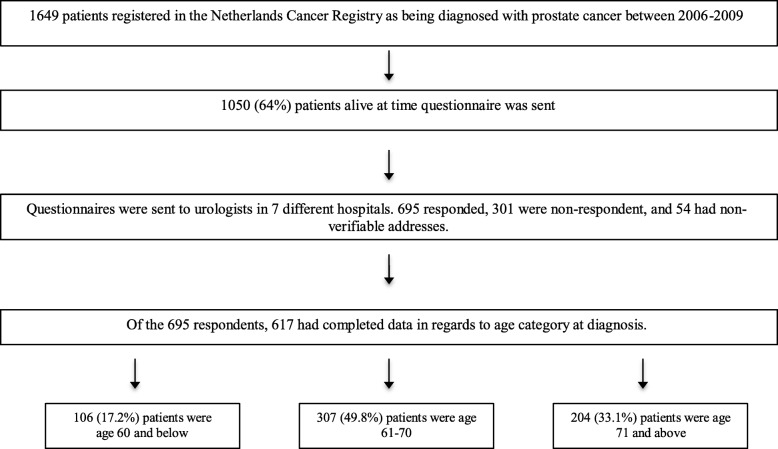
Narrowing of population of interest based on availability and completeness of data per individual

We requested patient data from the Patient Reported Outcomes Following Initial Treatment and Long-Term Evaluation of Survivorship (PROFILES) database to conduct a secondary data analysis. PROFILES collects data from the patients sampled from the Netherlands Cancer Registry (NCR). This data was collected in October 2011. PROFILES is a data registry designed to address the physical and psychosocial impact of cancer from a population of cancer survivors [10]. Data from PROFILES are available for non-commercial scientific research purposes (www.profilesregistry.nl), subject to study question, privacy, and confidentiality restrictions, and registration. Participants of the study live in the southern region of the Netherlands and were sampled from the NCR [11]. These individuals provided written consent to participate in the study and were invited to complete a questionnaire, which asked various questions pertaining to HRQL. Of 1649 patients diagnosed between 2006-2009, 1050 were still alive at the time of the HRQL collection. Of the remaining 1050 valid individuals, 695 responded to the invitation to participate in HRQL collection, 301 did not and 54 had non-verifiable addresses. Of the remaining 695 participants, 617 individuals had complete data in regards to the HRQL scales and were analyzed in the current study (Fig. 1).

### Study measures

#### European Organization for Research of Cancer quality of life questionnaire

Health-related quality of life was assessed using the European Organization for Research and Treatment of Cancer Quality of Life Questionnaire (EORTC-QLQ-C30) [12]. This self- reported questionnaire has 30 items related to global health status/quality of life, based on five functional scales and three symptom scales, to six single items on symptoms and financial impact [9]. This particular study assessed various scales including: global health, physical functioning, role functioning, emotional functioning, cognitive functioning, and social functioning scales (Table 1). Scores from each scale range from 0-100, with a higher score indicating a higher health-related quality of life. In addition to these scales, negative outcomes were also measured, including parameters such as fatigue, nausea, and pain, and prostate-related symptoms (Table 1). Prostate-related symptoms were evaluated using the EORTC QLQ-PR25 questions which focus on sexual activity and urinary, bowel, and hormonal symptoms. Lower scores for sexual activity and higher scores for urinary, bowel, and hormonal scales indicated increased severity of symptoms.

**Table 1:** Division of QOL scales into positive vs. negative measures

Positive Outcomes	Negative Outcomes
Global Health scale	Fatigue scale
Physical Functioning scale	Nausea scale
Role Functioning scale	Pain scale
Emotional Functioning scale	Dyspnea scale
Cognitive Functioning scale	Insomnia scale
Social Funtioning scale	Appetite scale
	Constipation scale
	Diarrhea scale

#### Demographic and clinical information

Socio-demographic and clinical data were also contained in the PROFILES database. Age at diagnosis was categorized as: less than and including 60 years of age, 61 to 70 years of age, and 71 years of age and older. Gleason score was categorized as low [2–6], medium [7], and high [8–10]. Other relevant patient information included the stage of cancer, ranging from I-IV, and the time since diagnosis, ranging in categories from less than 2 years to greater than 6 years. Treatments chosen included: prostatectomy, radiation therapy (external beam radiation, brachytherapy), hormonal therapy, and watchful waiting/active surveillance. Comorbidities, adapted from the Self- Reported Comorbidity Questionnaire, including heart conditions, stroke, hypertension, asthma, bronchitis, chronic obstructive pulmonary disease (COPD), diabetes, ulcers, kidney disease, liver disease, anemia, blood conditions, thyroid disease, depression, arthritis, backache, and rheumatism, were noted [13]. Highest education level was categorized as: lower (primary), secondary (high school and vocational), and university. Current marital status was assessed as married/cohabiting, and divorced/separated, widowed, and never married/never cohabiting. To simplify, categories were combined to reflect if individuals were coupled (married) or uncoupled (not married). Smoking behavior reflected current smokers, former smokers, and those who never smoked. Socioeconomic status (SES) was determined by an area-level indicator constructed by Statistics Netherlands [14]. Dutch postal codes were each assigned a value dependent on aggregated individual fiscal data on monetary home value and household income. The SES categories were then divided into low (deciles 1-3), medium (deciles 4-7), or high (deciles 8-10) [15]. Of the original data set containing 1649 individuals, 40 were characterized as residing in care-providing institutions. Ultimately these individuals were not included in the analysis because this category does not accurately reflect SES.

### Statistical analyses

To examine the relationship between age and quality of life, age category at diagnosis was chosen as the main independent variable. Dependent variables were each of the EORTC- QLQ-C30 and EORTC QLQ-PR25 scales, divided into positive and negative outcomes. The positive measures of health- related quality of life included global health, physical functioning, role functioning, emotional functioning, cognitive functioning, and social functioning. Negative outcomes included fatigue, nausea, pain, dyspnea (difficulty in breathing), insomnia, appetite, constipation, and diarrhea. Prostate-related symptoms were also analyzed as negative outcomes.Chi-squared tests (Fisher’s exact test where appropriate for cells with < 5 observations) and Kruskal-Wallis tests were performed to examine unadjusted relationships between specific age categories and demographics or other discrete variables. We conducted median regression modeling of the scales that showed some evidence of unadjusted associations with age group (i.e., *p* < 0.10) by the Kruskal-Wallis test. These models, controlling for BMI, marital status, time since diagnosis, comorbidities (heart condition), Gleason score, and treatment (prostatectomy) were constructed to adjust for potential confounders and better characterize the relationships between age category at diagnosis and each modeled health-related quality of life and prostate symptom scales. Four scales, (dyspnea, appetite, constipation, and finance) showed unadjusted associations with age group, but had at least 85% of respondents reporting zero values. Adjusted median regression models could not be fitted to these scales. The criterion for statistical significance was *p* < 0.05.

## Results

Figure 1 displays the study population, and Table 2 details demographic information and descriptive details. Eighteen percent of respondents (*N* = 106) were < age 61, 50% were age 61 to 70 (*n* = 307), and 33% were age 71+ (*N* = 204). Many demographic and clinical factors differed by age group. Body mass index (BMI, *p* = 0.032) and the proportion of men who were married (*p* = 0.004), college educated (< 0.001), current smokers (< 0.001), currently employed (< 0.001) or high SES (*p* = 0.002) were lowest among older men (age 71+). Higher Gleason score (*p* = 0.003) and comorbidities (specifically heart conditions (*p* = 0.006), diabetes (*p* = 0.041), kidney disease (*p* = 0.021), and anemia (*p* = 0.040)) were significantly more common among older men. Older men were most likely to be treated by hormone therapy or active surveillance/watchful waiting. Among younger men (age < 61), there was a greater number of years since diagnosis (*p* < 0.001) and they were most likely to be treated by prostatectomy (*p* < 0.001).

**Table 2:** Demographic characteristics of the PROFILES Cohort

Variable of Interest		All patients *N* = 617	Age Category at Diagnosis			*p*-value
			< 61 years *N* = 106	61–70 years *N* = 307	71+ years *N* = 204	
Body Mass Index [1st, 3rd quartiles]		26.3 [24.2, 28.3]	26.8 [24.8, 28.4]	26.2 [24.3, 28.4]	25.8 [23.9, 27.7]	**0.032**
Marital Status	Not married	97 (15.8)	12 (11.3)	39 (12.8)	46 (22.8)	**0.004**
	Married/Co-habiting	516 (84.2)	94 (88.7)	266 (87.2)	156 (77.2)	
Education	Lower	85 (14.0)	4 (3.8)	36 (11.8)	45 (22.5)	**<0.001**
	Secondary	366 (60.1)	70 (66.7)	176 (57.9)	120 (60.0)	
	University, higher	158 (25.9)	31 (29.5)	92 (30.3)	35 (17.5)	
Smoking Status	Never	140 (22.8)	34 (32.4)	64 (20.9)	42 (20.9)	**<0.001**
	Former	399 (65.1)	48 (45.7)	209 (68.1)	142 (70.7)	
	Current	74 (12.1)	23 (21.9)	34 (11.1)	17 (8.5)	
Currently Employed	No	512 (85.8)	56 (52.8)	274 (90.7)	182 (96.3)	**<0.001**
	Yes	85 (14.2)	50 (47.2)	28 (9.3)	7 (3.7)	
SES (%)	Low	98 (16.7)	15 (14.7)	35 (12.0)	48 (24.7)	**0.002**
	Medium	238 (40.5)	39 (38.2)	120 (41.1)	79 (40.7)	
	High	252 (42.9)	48 (47.1)	137 (46.9)	67 (34.5)	
Gleason Score (%)	2–6	329 (55.0)	63 (61.8)	178 (60.1)	88 (44.0)	**0.003**
	7	175 (29.3)	29 (28.4)	75 (25.3)	71 (35.5)	
	8–10	94 (15.7)	10 (9.8)	43 (14.5)	41 (20.5)	
Stage of Cancer (%)	I	292 (47.9)	57 (54.8)	154 (50.3)	81 (40.5)	0.121
	II	208 (34.1)	34 (32.7)	102 (33.3)	72 (36.0)	
	III	97 (15.9)	11 (10.6)	45 (14.7)	41 (20.5)	
	IV	13 (2.1)	2 (1.9)	5 (1.6)	6 (3.0)	
Time since Diagnosis (years, %)	< 2	10 (1.8)	1 (1.0)	5 (1.7)	4 (2.2)	**<0.001**
	2–4	280 (49.0)	35 (36.1)	143 (49.7)	102 (54.8)	
	4–6	272 (47.6)	54 (55.7)	139 (48.3)	79 (42.5)	
	> 6	9 (1.6)	7 (7.2)	1 (0.4)	1 (0.5)	
Comorbidities (%)	Heart Condition	124 (22.5)	15 (14.7)	56 (20.4)	53 (30.3)	**0.006**
	Stroke	13 (2.5)	0 (0.0)	9 (3.4)	4 (2.6)	0.174
	Hypertension	207 (37.6)	35 (34.7)	106 (37.9)	66 (38.8)	0.783
	Asthma, Bronchitis, COPD	71 (13.2)	7 (7.0)	40 (14.6)	24 (14.6)	0.128
	Diabetes	85 (15.7)	11 (10.9)	38 (14.0)	36 (21.3)	**0.041**
	Ulcer	10 (1.9)	1 (1.0)	5 (1.9)	4 (2.5)	0.681
	Kidney Disease	16 (3.0)	2 (2.0)	4 (1.5)	10 (6.1)	**0.021**
	Liver Disease	2 (0.4)	0 (0.0)	2 (0.8)	0 (0.0)	0.693
	Anemia/Blood Condition	25 (4.7)	2 (2.0)	10 (3.8)	13 (8.2)	**0.040**
	Thyroid Disease	14 (2.6)	2 (2.0)	4 (1.5)	8 (4.9)	0.092
	Depression	42 (8.0)	12 (11.9)	20 (7.5)	10 (6.3)	0.251
	Arthritis	141 (26.1)	20 (19.6)	69 (25.4)	52 (31.3)	0.098
	Backache	150 (28.0)	29 (28.4)	77 (28.2)	44 (27.5)	0.983
	Rheumatism	42 (8.1)	7 (7.1)	24 (9.1)	11 (6.9)	0.674
Treatment Type (%)	Prostatectomy	224 (36.7)	63 (60.0)	123 (40.5)	38 (18.8)	**<0.001**
	Radiation	278 (45.5)	41 (39.1)	148 (48.7)	89 (44.1)	0.204
	Hormone Therapy	126 (20.6)	15 (14.3)	54 (17.8)	57 (28.2)	**0.004**
	Active Surveillance/Watchful Waiting	27 (4.4)	5 (4.8)	12 (4.0)	10 (5.0)	0.850

The bolded *p*-values are significant (< 0.05)

Table 3 presents the results of non-parametric Kruskal-Wallis tests to examine scores for differences in HRQL and prostate symptoms by age group. Even though the age groups have the same or almost the same quartiles (1st, 2nd (median) and 3rd) for many of the scales in our sample, the test is sensitive to differences elsewhere in distributions of these scales. Significant findings for these scales suggest that at least one of the age-defined populations tends to have lower scale values. We observed that older men (age 71+) tended to have lower values for Global Health (*p* = 0.006), physical functioning (*p* < 0.001) and cognitive functioning (*p* = 0.010). Their scores were higher for fatigue (*p* = 0.003), appetite (*p* = 0.002), and constipation (*p* < 0.001). Men age < 61 were more likely to be impacted financially compared to older men (*p* = 0.002).

**Table 3:** Health-related quality of life and prostate symptom scales by age category

Median Score [1st, 3rd quartiles]		< 61 years	61–70 years	71+ years	*p*-value
Health Related Quality of Life	Global Health Scale	83.3 [66.7, 91.7]	83.3 [66.7, 91.7]	83.3 [66.7, 83.3]	**0.006**
	Physical Functioning	93.3 [80.0, 100]	93.3 [80.0, 100]	80 [66.7, 93.3]	**<0.001**
	Role Functioning	100 [66.7, 100]	100 [66.7, 100]	100 [66.7, 100]	0.053
	Emotional Functioning	100 [75.0, 100]	100 [83.3, 100]	100 [83.3, 100]	0.517
	Cognitive Functioning	100 [83.3, 100]	100 [83.3, 100]	83.3 [75.0, 100]	**0.010**
	Social Functioning	100 [83.3, 100]	100 [83.3, 100]	100 [83.3, 100]	0.095
	Fatigue Scale	11.1 [0, 33.3]	11.1 [0, 22.2]	22.2 [0, 33.3]	**0.003**
	Nausea Scale	0 [0, 0]	0 [0, 0]	0 [0, 0]	0.722
	Pain Scale	0 [0, 33.3]	0 [0, 16.7]	0 [0, 33.3]	0.592
	Dyspnea Scale	0 [0, 33.3]	0 [0, 33.3]	0 [0, 33.3]	0.085
	Insomnia Scale	0 [0, 33.3]	0 [0, 33.3]	0 [0, 33.3]	0.851
	Appetite Scale	0 [0, 0]	0 [0, 0]	0 [0, 0]	**0.002**
	Constipation Scale	0 [0, 0]	0 [0, 0]	0 [0, 0]	**<0.001**
	Diarrhea Scale	0 [0, 0]	0 [0, 0]	0 [0, 0]	0.834
	Financial Scale	0 [0, 0]	0 [0, 0]	0 [0, 0]	**0.002**
Prostate Symptoms	Sexual Activity	33.3 [16.7, 50.0]	33.3 [0, 33.3]	16.7 [0, 33.3]	**<0.001**
	Urinary Symptoms	12.5 [4.2, 20.8]	16.7 [8.3, 28.6]	16.7 [8.3, 29.2]	**0.030**
	Bowel Symptoms	0 [0, 0]	0 [0, 8.3]	0 [0, 8.3]	**0.011**
	Hormonal Symptoms	11.1 [5.6, 16.7]	11.1 [0, 16.7]	8.3 [0, 16.7]	0.384
	Urinary aid problems	0 [0, 33.3]	0 [0, 33.3]	0 [0, 33.3]	0.791

The bolded *p*-values are significant (< 0.05)

Prostate symptoms also varied by age. Sexual activity was lowest for men age 71+ (*p* < 0.001) while urinary (*p* = 0.030) and bowel symptom scores (*p* = 0.011) were higher for these men.

Among the numerous scales showing some unadjusted association with age group, only two scales demonstrated significant differences between prostate cancer patients age 71+ compared to the youngest group (age < 61) after multivariable adjustment. On average, the oldest patients experienced an 8.3-point lower median physical functioning score (β = − 8.3; 95% CI = − 13.9, − 2.8; *p* = 0.003) and 16.7 point lower median sexual activity score (β = − 16.7; 95% CI = − 24.7, − 8.6; *p* < 0.001) while controlling for BMI, marital status, time since diagnosis, comorbidities (heart condition), Gleason score, and treatment (prostatectomy).

## Discussion

This study examined relationships between the age at diagnosis and health-related quality of life parameters. In univariate analyses, younger men were found to have more positive outcomes and decreasing negative outcomes with respect to many (10/20) of the HRQL scales. We observed a significant decrease in global health for older men (age 71+), which was the main scale used for the reference populations. However, we found no significant difference in global health after adjusting for possible confounders. Multivariable models indicated that only physical functioning and sexual activity remain statistically significant. After multivariable analysis, young men still reported more positive scores on the physical functioning and sexual activity. These outcomes were the only parameters that were significantly worse among older patients suggesting that when other factors (co-morbidities, marital status, tumor aggressiveness, and treatment type) were taken into consideration, older patients generally had HRQL that was comparable to that of younger patients.

But, we must be cautious in our interpretation that these age-related findings are only specific to prostate cancer survivors. In fact, changes in physical functioning and sexual activity with advancing age may be a consequence of aging. For example, a study by Mols, et al. compared physical functioning with cancer survivors and an age-matched normative population and did not find significant differences [16]. In our study, we were not able to examine underlying factors leading to better physical and sexual functioning among younger patients. However, it is possible that younger men with prostate cancer are healthier at the time of diagnosis and/or that younger men fare better through treatment compared to older men. Generally, younger age is associated with a higher likelihood of sexual activity and greater physical functioning [17–19]. Poor physical functioning also serves as a barrier to engaging in sexual activity [17].

Several studies have examined the EORTC QLQ-C30 in normative populations to determine appropriate reference values and the validity of the questionnaire. van de Poll-Franse and colleagues examined the EORTC QLQ-C30 in the general Dutch population (including both men and women), finding that increased age led to declining functional health (all scales except for emotional functioning) [20]. They also discovered that older age led to worse pain and fatigue scores. Hinz et al. examined the use of the EORTC QLQ-C30 in the general German population in 2011, finding that the older populations had a significantly lower global health score than younger populations [21]. A study by Velenik et al. examining HRQL measures in the EORTC QLQ-C30 in the general Slovenian population found that scores were significantly affected by gender, age, and social class; mean scores decreased with increasing age [22]. Comparing our results in Dutch prostate cancer survivors to these reference values of normative populations, age was a significant factor leading to decreased physical functioning.

Among the limited studies using similar European questionnaires in prostate cancer patients and/or survivors, the impact of age at diagnosis is not fully elucidated. Dabrowska-Bender and colleagues looked at subjective quality of life in Polish prostate cancer patients diagnosed between the ages of 51-84 using the EORTC QLQ-C30 questionnaire, and did not find significant differences between the quality of life scores in differing age groups [23]. Another study examining our population of interest, Dutch prostate cancer survivors, found that younger men reported less bodily pain and higher physical functioning on the 36-item short form survey (SF-36) [16]. A previous review by Blank and Bellizzi observed that in relation to aging and the survivorship experience, younger cancer survivors experienced trajectories of greater impacts (both negative and positive) and were more likely to make health behavior changes than survivors who are older at diagnosis [24]. The review also noted that younger survivors experienced fewer comorbidities and a greater level of physical functioning and health. However, survivors that were older when diagnosed were less likely to change their behavior. Blank and Bellizzi reasoned that this may have resulted in a higher quality of life, as a push towards earlier detection may, for some younger individuals, lead to increased stress and an overall negative psychological outlook [24]. Results are still unclear whether the long-term effects on HRQL are the same for younger and older survivors. More research will be needed to solidify any conclusions.

Sexual dysfunction, a loss of pleasure and diminution in sexual ability and activity, is a common long-term consequence of prostate cancer treatment. Following treatment, many men and their partners develop sexual dysfunction in response to erectile dysfunction and other side effects [25]. Indeed, the men in our study who were diagnosed at a younger age reported fewer symptoms related to sexual activity than men who were diagnosed later in life. Sexual dysfunction influences social relationships and quality of life, and while a substantial amount of research has documented this problem in prostate cancer survivors [26–29], less is known about the lived experiences of the men and the impact it has on their partners and spouses [30, 31].

Our findings also speak to the need for integration of geriatric oncology into clinical practice, as the health needs of older patients are not the same as those of younger patients. Since 2005, the comprehensive geriatric assessment has been advocated for senior adult oncology patients, defined as those aged 70 years or older [32]. Comprehensive screening of older patients will identify problems in sexual functioning as well as characterize general health status, so an appropriate plan of treatment can be formulated for all patients. Clinicians and other health care providers should be aware of the impact of prostate cancer treatment on long-term sexual health, particularly among their patients of various age groups, and ensure that patients are connected to the appropriate resources to address their concerns. Appropriate social support and survivorship care plans may facilitate communication about functional and sexual well-being of aging prostate cancer survivors [17, 18, 33]. With a growing number of prostate cancer survivors, it is important to monitor the HRQL of patients as they continue post-treatment care.

### Study limitations

Limitations existed within the analysis, which may have contributed to or detracted from the significant associations found. The study population of men was reduced to ensure completeness of data. This could have excluded certain data outliers that may have resulted in otherwise unremarkable trends, resulting in selection bias. When we compared the original sample to the study sample, we found significant differences between the two populations in regards to age category at diagnosis (*p* <0.001), with the original population having an increased number of older individuals. This suggests that the results obtained may not be applicable to widespread populations in this preliminary stage. In particular, since older age is generally associated with poorer HRQL, the results may underestimate the effects of old age on HRQL. The presence of non-respondents and unverified addresses indicates that not all members of the study sample were evaluated. Additionally, the cross-sectional design contains limitations of its own because causality cannot be readily inferred. Since the data does not consider patients from a longitudinal standpoint, no comparison can be made between previous and current HRQL in individuals. Relating to this, baseline data were not available for this population so there was no means of comparison between previous and current HRQL.

### Study strengths

Completeness of the data contributed to more robust associations between the different variables. Only those who answered questions pertaining to the health-related quality of life scales were included. To our knowledge, this is the first study to examine the effects of age at diagnosis on HRQL of Dutch prostate cancer survivors using the EORTC QLQ-C30 and EORTC-QLQ-PR25 scales.

## Conclusions

Prostate cancer patients with a younger age at diagnosis reported the highest health- related quality of life. Men diagnosed at a younger age showed higher levels of physical functioning and sexual activity. Results suggest a possible reevaluation of screening recommendations to acknowledge patient age as a factor contributing to health-related quality of life outcomes for prostate cancer survivors.

## Abbreviation

HRLQ: Health-related quality of life
